# Seh1 targets GATOR2 and Nup153 to mitotic chromosomes

**DOI:** 10.1242/jcs.213140

**Published:** 2018-05-01

**Authors:** Melpomeni Platani, Itaru Samejima, Kumiko Samejima, Masato T. Kanemaki, William C. Earnshaw

**Affiliations:** 1 Wellcome Trust Centre for Cell Biology, Institute of Cell Biology, University of Edinburgh, Edinburgh EH9 3BF, UK; 2 Division of Molecular Cell Engineering, National Institute of Genetics, ROIS, and Department of Genetics, SOKENDAI, Yata 1111, Mishima, Shizuoka 411-8540, Japan

**Keywords:** Nuclear pore complex, NPC, Seh1, Nup153, Kinetochore microtubules, GATOR2, Chromosome, mTOR, CPC

## Abstract

In metazoa, the Nup107 complex (also known as the nucleoporin Y-complex) plays a major role in formation of the nuclear pore complex in interphase and is localised to kinetochores in mitosis. The Nup107 complex shares a single highly conserved subunit, Seh1 (also known as SEH1L in mammals) with the GATOR2 complex, an essential activator of mTORC1 kinase. mTORC1/GATOR2 has a central role in the coordination of cell growth and proliferation. Here, we use chemical genetics and quantitative chromosome proteomics to study the role of the Seh1 protein in mitosis. Surprisingly, Seh1 is not required for the association of the Nup107 complex with mitotic chromosomes, but it is essential for the association of both the GATOR2 complex and nucleoporin Nup153 with mitotic chromosomes. Our analysis also reveals a role for Seh1 at human centromeres, where it is required for efficient localisation of the chromosomal passenger complex (CPC). Furthermore, this analysis detects a functional interaction between the Nup107 complex and the small kinetochore protein SKAP (also known as KNSTRN).

## Abstract (precis)

**Highlighted Article:** The nucleoporin Seh1 is essential for the association of both the GATOR2 complex and the nucleoporin Nup153, but not the Nup107 complex, with mitotic chromosomes.

## INTRODUCTION

The nuclear pore complex (NPC) is a large macromolecular assembly anchored at the nuclear envelope that allows transport between the nucleus and the cytoplasm during interphase (Hurt and Beck, 2015). Recently, the nucleoporins (components of the NPC) have been implicated in a number of processes unrelated to nucleocytoplasmic transport (Hetzer and Wente, 2009; Knockenhauer and Schwartz, 2016; Strambio-De-Castillia et al., 2010; Wozniak et al., 2010). An initial observation suggesting that there could be an alternative function for the nucleoporins was finding that a subset of NPC components localised to the kinetochores of mitotic chromosomes (Belgareh et al., 2001; Hetzer and Wente, 2009; Joseph et al., 2002; Loiodice et al., 2004; Strambio-De-Castillia et al., 2010; Zuccolo et al., 2007).

Kinetochores are complex macromolecular assemblies on centromeric DNA that orchestrate chromosome segregation by providing attachment and signalling sites for spindle microtubules (Cheeseman, 2014; Samejima et al., 2017). The nucleoporins that localise to kinetochores are members of the evolutionarily conserved Nup107 complex (also known as the Y complex), a core structural scaffold of nuclear pores in interphase located on both faces of the NPC. In vertebrates, the Nup107 complex is composed of ten different nucleoporins: Nup160, Nup133, Nup107, Nup96, Nup85, Nup43, Nup37, Sec13, Seh1 (also known as SEH1L) and Elys (also known as AHCTF1) (Knockenhauer and Schwartz, 2016). The Nup107 complex plays a crucial role in NPC assembly, mRNA export and cell differentiation (González-Aguilera and Askjaer, 2012; Harel et al., 2003; Vasu et al., 2001; Walther et al., 2003). Nup107 complex components remain associated together throughout mitosis and are among the earliest nucleoporins recruited onto chromatin during nuclear envelope reformation at the end of cell division (Belgareh et al., 2001).

Previous approaches to study the mitotic function of the Nup107 complex have used immunodepletion in mitotic *Xenopus* egg extracts, where a role of the Nup107 complex in spindle assembly was revealed (Mishra et al., 2010; Orjalo et al., 2006; Yokoyama et al., 2014). In other RNAi studies in human cells and *C. elegans*, different members of the Nup107 complex were implicated in kinetochore composition and disassembly, kinetochore microtubule attachment strength and chromosome segregation (Hattersley et al., 2016; Platani et al., 2009; Rasala et al., 2006; Rodenas et al., 2012; Zuccolo et al., 2007).

Seh1 is a member of both the Nup107 complex and the unrelated GATOR2 complex, which has been reported to play a role in mTORC1 regulation as an inhibitor of the Rag GTPases (Bar-Peled et al., 2013; Panchaud et al., 2013a). mTORC1 kinase coordinates cell growth and proliferation in response to nutrient supply. A few previous studies have revealed a link between mTORC1 regulation and mitosis (Astrinidis et al., 2006; Halova and Petersen, 2011; Renner et al., 2010). More recently, the regulation of the mTORC1 kinase by the GATOR2 complex was linked to the spatiotemporal activation of Aurora A and Plk1 at centrosomes during mitosis (Platani et al., 2015).

In this study, we used a chemical genetics approach to acutely and rapidly control Seh1 protein levels in cells by means of the auxin-inducible degron (AID) system. We generated cell lines in which Seh1 is conditionally degraded in a few hours. In this way, we could analyse the mitotic function of Seh1 separately from its interphase function, thus minimising defects arising from complications in nucleocytoplasmic transport or inhibition of the GATOR2 complex during interphase. We also combined quantitative proteomics with DT40 cell genetics by using conditional knockout (KO) cell lines to look at the response of other protein complexes on mitotic chromosomes to acute depletion of Seh1. We confirmed a previous report that Seh1 functions in chromosome alignment and segregation by regulating the centromeric localisation of Aurora B in human cells. We went on to show that the GATOR2 activator of mTORC1 kinase associates with mitotic chromosomes and that this association is highly dependent on Seh1. Core structural kinetochore components and other members of the Nup107 complex were unaffected by Seh1 degradation, while the unrelated nucleoporin Nup153, which is not a member of the Nup107 complex and was not previously known to associate with metaphase mitotic chromosomes, was highly affected. Finally, we identified a previously unsuspected functional interaction between Seh1 and small kinetochore-associated protein SKAP (also known as KNSTRN).

## RESULTS

### Use of the AID system to establish an HCT116 cell line from which Seh1 is rapidly removed

**Fig. 1.**
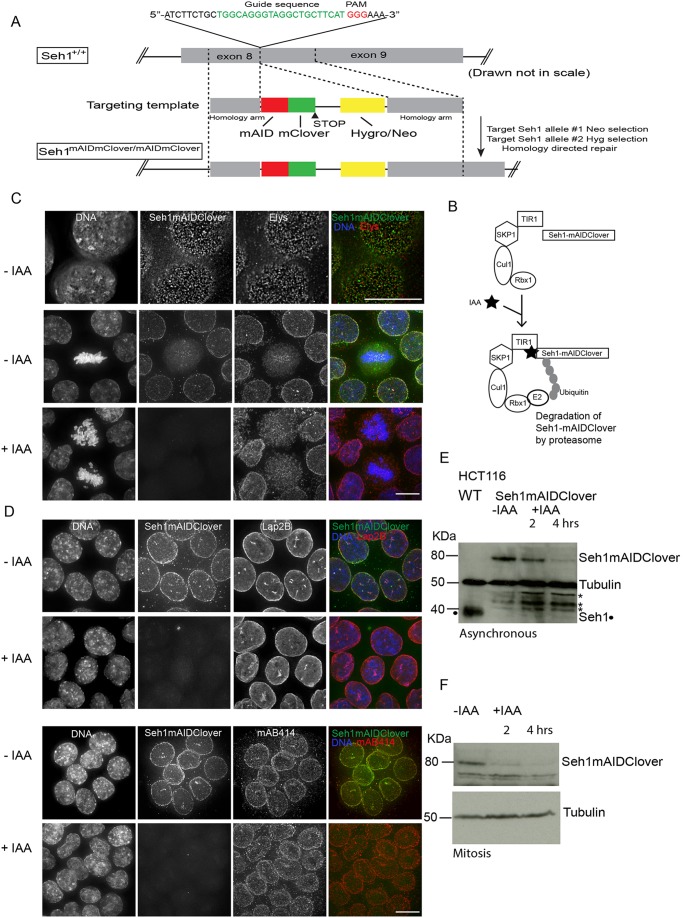
**Establishment of a rapid auxin-inducible Seh1 degradation system.** Establishment of Seh1–mAIDmC HCT116 cells expressing OsTIR1 and Seh1–mAID–mClover. (A) Strategy for the insertion of the mAID–mClover coding sequence just upstream of the termination codon of the human *Seh1* gene. A schematic diagram of the guide (g)RNA/targeting template-targeting site at *Seh1* exon 8 is shown. The gRNA sequence is shown in green and PAM motif in red. The targeting template contains 500 bp homology arms that flank the double-strand break site and a mAID–mClover Hygro or/Neo cassette. The genomic configuration expected to be generated via homology-directed repair is shown underneath. (B) Schematic illustration of the AID system. OsTIR1 is part of the SCF^OsT^^I^^R1^ E3 ligase complex, and works together with an endogenous E2. Upon addition of indole-3-acetic acid (IAA), Seh1 protein tagged with mAID is polyubiquitylated and degraded. (C,D) The subcellular localisation of Seh1–mAID–mClover (green) is shown in interphase or mitotic cells in the absence or presence of IAA (4 h). Cells were counterstained for the Nup107 complex member (Elys, red), a nuclear envelope component (Lap2B) (red), and FG repeat nucleoporins (mAB414) (red). DNA is shown in blue. (E) Immunoblots of total cell lysates of from the HCT116 OsTIR1 parental cell line and Seh1-mAIDmC cell line in the absence (−IAA) or presence (+IAA) of auxin during interphase (E) or mitosis (F) probed using anti-Seh1 and anti-tubulin. Seh1–mAIDmC cells were synchronised in mitosis with Monastrol and were either mock treated or treated with IAA for the corresponding amount of time still in the presence of Monastrol. Tubulin serves as a loading control. *non-specific band (Seh1 is marked with a black dot). Scale bars: 10 µm.

We used degron tagging of Seh1 to rapidly deplete this protein in order to study its role in mitosis. The AID system enables the rapid degradation of proteins of interest in a variety of eukaryotic cells (Nishimura et al., 2009; Samejima et al., 2014). CRISPR-Cas9 gene editing technology allows efficient knock-in of specific sequences (such as tags) at a targeted locus (Cong et al., 2013; Mali et al., 2013). We employed both of these technologies (Natsume et al., 2016) to establish an HCT116 cell line in which endogenous Seh1 protein is tagged at the C-terminus with mini AID-monomeric Clover (mAIDmC) (Fig. 1A,B). This allowed the visualisation of the tagged endogenous Seh1 through mClover and the conditional degradation of Seh1 upon addition of Auxin (indole-3-acetic acid; IAA) to the medium (Fig. 1C). As expression of Seh1–mAIDmC was under the control of the endogenous promoter for the Seh1 gene, the levels of Seh1–mAIDmC were similar to the Seh1 levels in the parental HCT116 cell line (Fig. 1E). Western blot analysis confirmed the depletion of Seh1–mAIDmC within 2 h after the addition of Auxin in mitotic cells (Fig. 1F). The efficiency of Seh1 degradation at 4 h following IAA addition is decreased in asynchronous cultures, probably as a result of incorporation of Seh1 into the NPC plus its ongoing protein synthesis (Fig. 1E).

Seh1–mAIDmC localisation was similar to that of the endogenous Seh1, showing the characteristic nuclear pore localisation in interphase and kinetochore localisation in mitosis. Seh1–mAIDmC colocalised with Elys, Nup133 and Nup107, three other members of the Nup107 complex both in interphase and mitosis (Fig. 1C; Figs S1A,B and S3A). Thus, the mAID-mCr tag did not detectably affect the behaviour of the endogenous Seh1 protein. Addition of IAA to the medium resulted in rapid loss of the protein from both interphase and mitotic cells (Fig. 1C). This only mildly decreased the kinetochore localisation of Elys, another member of the Nup107 complex. The localisation and levels of other unrelated nucleoporins (stained with mAB414, which recognises FG repeat nucleoporins) and nuclear membrane proteins (Lap2B) appeared to be unaffected (Fig. 1D).

Five independent Seh1–mAIDmC clonal cell lines generated as described in the Materials and Methods behaved in the same way regarding the localisation and rate of degradation of the Seh1–mAIDmC protein.

### Role of Seh1 in mitotic progression following rapid degradation

**Fig. 2.**
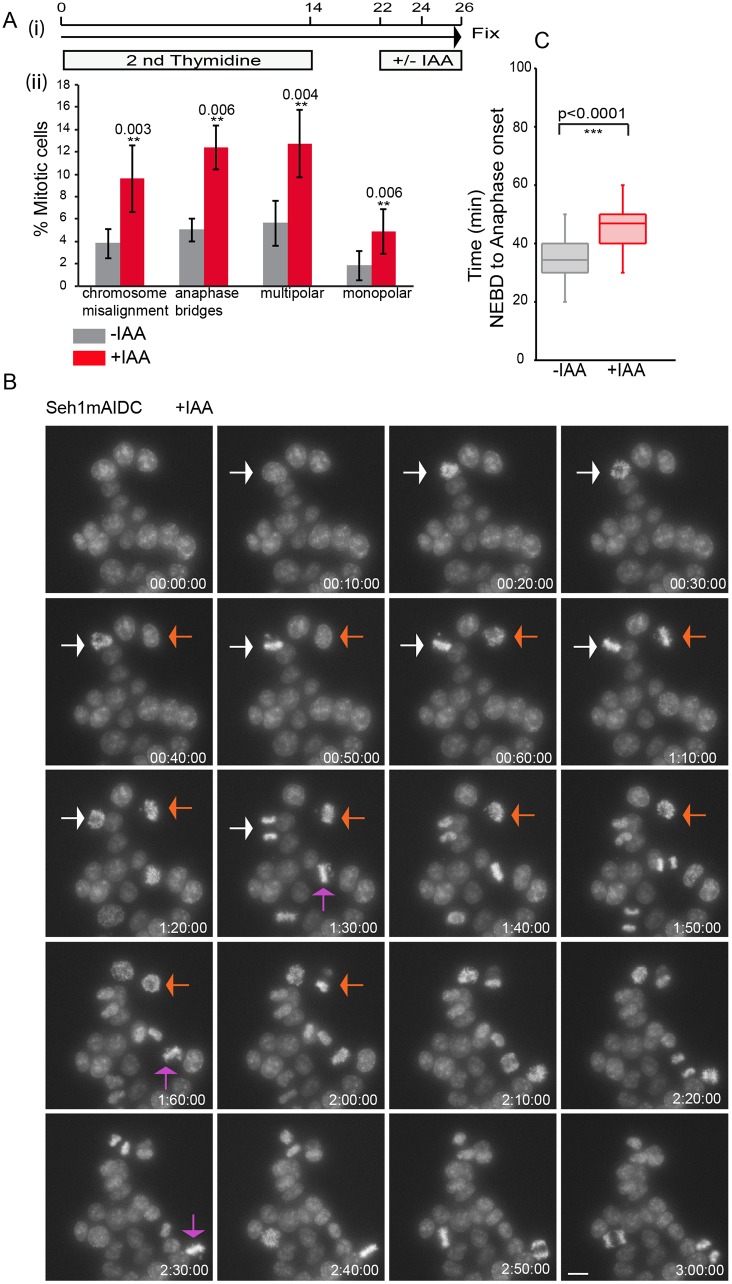
**Rapid Seh1 degradation affects mitotic progression.** (A) (i) Schematic diagram of the Seh1–mAIDmC cell line synchronisation protocol. Seh1–mAIDmC cells were incubated without (−IAA) or with auxin (+IAA) for 4 h prior to fixation and immunofluorescence analysis for microtubules and centromere markers. (ii) Quantification of observed mitotic defects. Results are mean± s.d. for *n*=500 (−IAA) and *n*=613 (+IAA) cells from three independent experiments. (B) Degradation of Seh1 affects mitotic progression. Selected maximum intensity projections from time-lapse images of Seh1–mAIDmC cells following addition of IAA showing mitotic defects (DNA stained with Hoechst 33342). Images were collected every 10 min over 3 h. Numbers indicate time in h:min:s. Arrows point to mitotic cells. (C) Mitotic progression box plots of anaphase onset with NEBD as *t*=0 in control (−IAA) (grey box) and Seh1-depleted cells (+IAA) (red box) from live-cell videos. *n*=169 (−IAA) and *n*=261 (+IAA) cells from three independent experiments. Statistical significance was determined by a two-tailed, unpaired *t*-test. Scale bar: 10 µm.

Seh1 plays important roles both in interphase and mitosis (Knockenhauer and Schwartz, 2016). Having previously described a requirement for Seh1 in mitotic progression (Platani et al., 2009), we wanted to examine the mitotic function of Seh1 separately from its interphase function. Seh1–mAIDmC cells were synchronised by means of a double thymidine block, and IAA was added for 4 h after release from the second block, during the transition from G2 to M phase. Addition of IAA resulted in an increased frequency of mitotic defects, including cells with chromosome misalignments, anaphase bridges, binucleation, multipolar or monopolar spindles (Fig. 2A, red bars; Fig. S3C). Detailed live-cell imaging of Seh1–mAIDmC cells following thymidine synchronisation and IAA addition revealed a small but highly reproducible delay in the timing from nuclear envelope breakdown (NEBD) to anaphase onset (from 36.3±11.1 min to 48±9.2 min; mean±s.d.). This mitosis was often accompanied by misaligned chromosomes in metaphase and lagging chromosomes in anaphase (Fig. 2B,C; Fig. S4A,B).

**Fig. 3.**
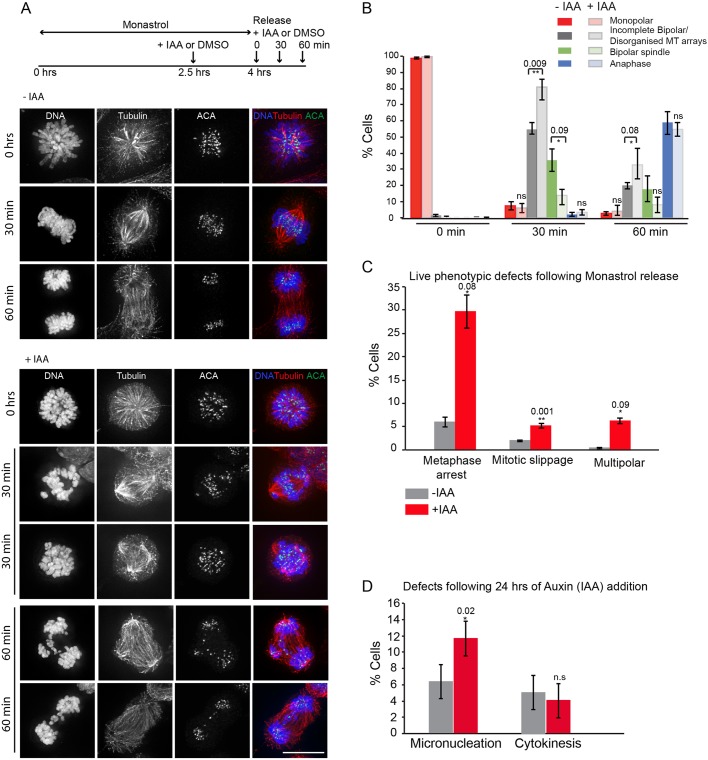
**Seh1 plays a role in mitotic progression.** (A) Schematic diagram of Seh1–mAIDmC cell line synchronisation with Eg5 inhibitor Monastrol prior to IAA addition or not. The drug was washed out with fresh medium prior to release in medium with or without IAA. Cells were fixed at the indicated time points and immunostained with anti-tubulin (red) and -ACA antibodies (green) and for DNA (blue). (B) Quantification of spindle and chromosome alignment in mock (−IAA) and Seh1-depleted (+IAA) cells at the indicated time points after Monastrol release. *n*=400 (−IAA) and *n*=400 (+IAA) cells. (C) Quantification of mitotic defects in mock (−IAA) (grey bars) and Seh1-depleted (+IAA) cells (red bars) from live-cell videos following Monastrol release. *n*=280 (−IAA) and *n*=295 (+IAA) cells. (D) Quantification of micronucleation and cytokinesis defects in mock (−IAA) (grey bars) and Seh1-depleted (+IAA) cells (red bars) following addition of IAA for 24 h. *n*=300 (−IAA) and *n*=300 (+IAA) cells. Results in all panels are from three independent experiments. *P<0.05; **P<0.01; ns, not significant (two-tailed, unpaired *t*-test). Error bars represent s.d. Scale bar: 10 µm.

To allow a quantitative measurement of spindle assembly and chromosome alignment defects, we examined the recovery of Seh1–mAIDmC cells from Monastrol in the presence or absence of IAA. Monastrol is an Eg5 (also known as KIF11) inhibitor that arrests cells in a state corresponding to prometaphase with monopolar spindles, but without affecting microtubule dynamics (Kapoor et al., 2000). At 30 min after Monastrol washout, about half of control cells (−IAA) formed a bipolar spindle, with 54% of cells still having a disorganised spindle. In contrast, 30 min after Monastrol washout, 82% of cells in the presence of IAA (+IAA) had either incomplete or tripolar spindles. Although progression to anaphase occurred by 60 min, it was often accompanied by the presence of chromatin bridges (Fig. 3A,B). Analysis of live metaphase cells in Seh1–mAIDmC cultures released from Monastrol in the presence of IAA (+IAA) showed a 6-fold increase, from 5% to 29%, in cells arrested in metaphase and not progressing to anaphase over the 1.5 h of image acquisition. The proportion of cells with mitotic slippage [exit from mitosis prior to spindle assembly checkpoint (SAC) satisfaction] increased from 2 to 5%, and those multipolar spindles increased to 5% (Fig. 3C). Finally, addition of IAA to the medium for longer periods of time (24 h) resulted in an increase of micronucleation to twice normal levels, from 6 to 12% (Fig. 3D).

**Fig. 4.**
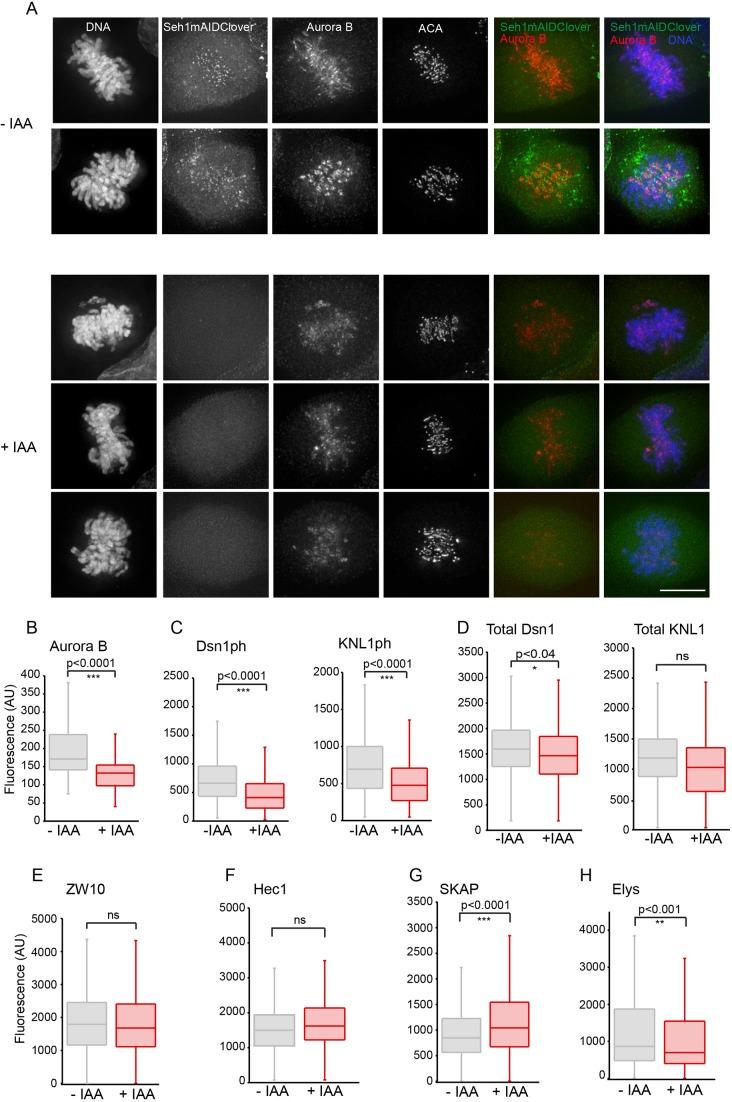
**Rapid degradation of Seh1 reduces centromeric levels of Aurora B while levels of outer kinetochore proteins are mostly unaffected.** (A) Mock (−IAA) and Seh1-depleted (+IAA) treated Seh1–mAIDmC cells were fixed and immunostained with anti-Aurora B (red) and -ACA (Cy5, grey) antibodies, and for DNA (blue). Seh1-mAID–mClover is green. (B) Quantification of Aurora B levels at centromeres in mock (−IAA), *n*=40, and Seh1-depleted (+IAA), *n*=40, cells. Aurora B levels at centromeres are reduced. (C) Quantification of Dsn1^ph^ and KNL1^ph^ levels at centromeres in mock (−IAA) (*n*=66, Dsn1^ph^; *n*=55, KNL1^ph^) and Seh1-depleted (+IAA) (*n*=63, Dsn1^ph^; *n*=54, KNL1^ph^) cells. Both Dsn1^ph^ and KNL1^ph^ levels are reduced. (D) Quantification of total Dsn1 and KNL1 levels at centromeres in mock (−IAA) (*n*=47, Dsn1; *n*=35, KNL1 cells) and Seh1-depleted (+IAA) (*n*=49, Dsn1; *n*=35, KNL1 cells). Quantification of kinetochore components ZW10 (*n*=30, −IAA; *n*=35, +IAA) (E), Hec1 (*n*=30, −IAA; *n*=30, +IAA) (F), SKAP (*n*=35, −IAA; *n*=35, +IAA) (G), Elys (Nup107 complex) (*n*=35, −IAA; *n*=35, +IAA) (H) in mock (−IAA) and Seh1-depleted (+IAA) cells. Fluorescence intensities are in arbitrary units (AU). Data are shown from three independent experiments. *P<0.05; **P<0.01; ***P<0.001; ns, not significant (two-tailed, unpaired *t*-test). Scale bar: 10 µm.

Micronuclei and lagging chromosomes often occur as a result of kinetochore–microtubule misattachments, either due to defects in error correction or spindle formation. Previous RNAi studies have found that Seh1-depleted cells show an impaired Aurora B localisation that is associated with severe defects in biorientation and organisation of the spindle midzone (Platani et al., 2009). To determine whether the molecular mechanism of the mitotic defects observed in Seh1–mAIDmC cells upon acute addition of IAA is consistent with previous studies, the levels and localisation of Aurora B were investigated following Monastrol arrest and release for 30 min in the presence (+) or absence (−) of IAA (Fig. 4). Upon degradation of Seh1 (+IAA), reduced levels of Aurora B were observed at centromeres relative to those in control (−IAA) cells (Fig. 4A,B). Aurora B phosphorylates multiple subunits of the KNL1–Mis12–NDC80 complex (KMN network) necessary for the tension-dependent fine-tuning of kinetochore-microtubule interactions (Welburn et al., 2010). Although we observed no significant changes in the localisation or total levels of KMN network members Dsn1 and KNL1 (Fig. 4D, Fig. S6E,F), the levels of DSN phosphorylated on S109 (Dsn1^S109ph^) and KNL phosphorylated on S24 (KNL1^Ser24ph^) were significantly reduced (Fig. 4C), consistent with reduced levels of Aurora B activity.

We also used indirect immunofluorescence to examine other outer kinetochore proteins following rapid and potent depletion of Seh1. To avoid effects on the kinetochore caused by microtubule binding, Seh1–mAIDmC cells were incubated in nocodazole prior to addition (or not) of IAA (Fig. S6A–D). These studies revealed a small, but consistent, decrease in the levels of kinetochore-associated nucleoporin Elys, another member of Nup107 complex, and a small but significant, increase in the levels of SKAP (Fig. 4G,H). We detected no changes in levels of the outer kinetochore proteins ZW10 and Hec1 (Fig. 4E,F).

### Dependency of chromosomal associated proteins on Seh1

**Fig. 5.**
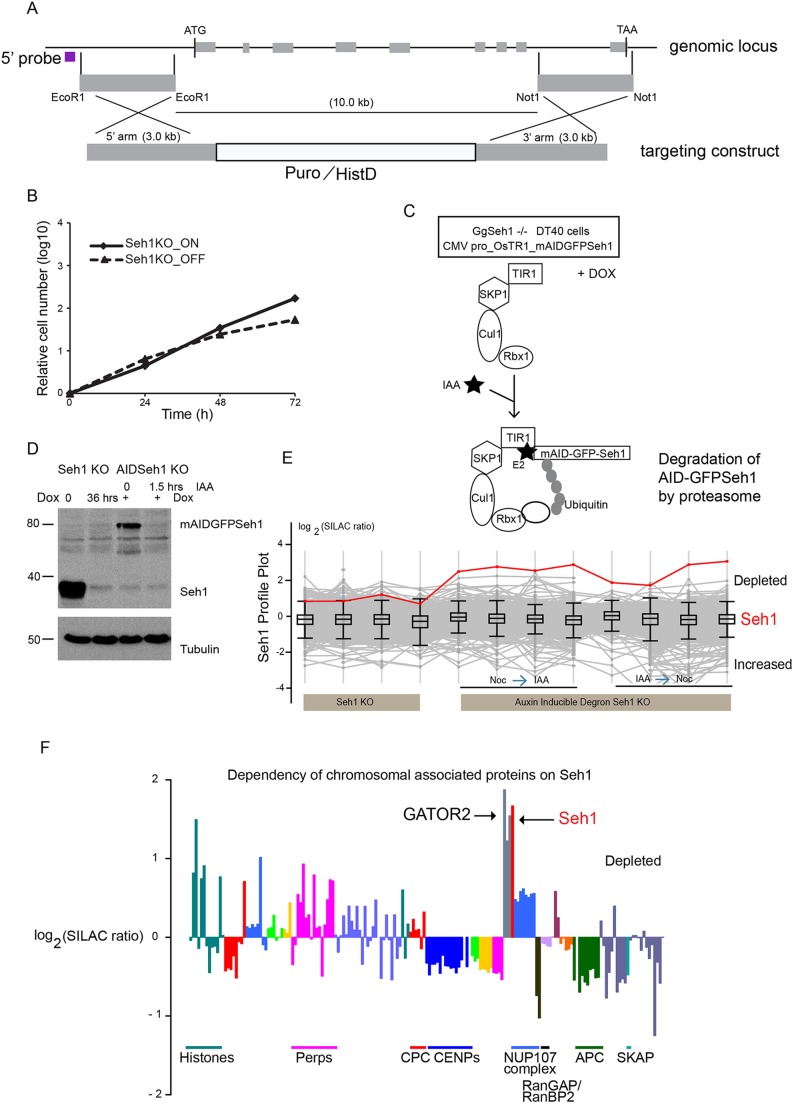
**Identification of chromosomal proteins dependent on Seh1 for their localisation as determined in a** **proteomics analysis.** (A) Schematic representation of the Seh1 genomic DT40 locus and targeting construct to create the Seh1 conditional KO cell line. Grey boxes represent exons. (B) Growth curves of DT40 Seh1 KO cells in the absence (Seh1KO_ON) or presence (Seh1KO_OFF) of doxycycline (Dox). (C) Schematic representation of the AID system used in DT40 cells. A plasmid encoding OsTIR1 and mAID–GFP–Seh1 was transfected into Seh1**^−/−^** conditional KO cells. F-box protein OsTIR1 binds to the endogenous SKP1 to form the SCF^OsTIR1^ complex. TIR1 binds to the mAID tag in the presence of IAA. SCF^OsTIR1^ ubiquitylates the mAID tag and promotes the degradation of the mAID–GFP–Seh1 protein by the proteasome. (D) Immunoblots of total cell lysates of Seh1 conditional KO in the absence or presence of Dox (36 h) and of AID-Seh1 conditional KO cells in the absence (−IAA) or presence (+IAA, 1.5 h) of IAA probed using anti-Seh1 antibody. Tubulin serves as a loading control. (E) Seh1 is highly depleted from chromosomes. Profile plots show the log_2_ (H/L) ratios of all proteins in all experiments (grey lines). The interquartile population of all proteins detected in each experiment is contained in the box. Whiskers extend to 1.5× the interquartile range from the edge of the box. The *y*-axis is labelled so that proteins that have a higher amount of chromosomes are plotted downwards and proteins that decrease are plotted upwards. The behaviour of Seh1 is shown as a red line. (F) Bar plot of log_2_ H/L ratios from Seh1 KO_OFF cell line. Chromosomal, NPC and kinetochore proteins are grouped according to protein complexes.

Quantitative proteomics combined with DT40 genetics is a valuable tool in understanding the behaviour of the entire mitotic chromosome proteome and protein complexes following the depletion of defined components (Samejima et al., 2015a). Having detected changes in centromeric levels of Aurora B, and small but consistent changes in SKAP and Elys, we decided to use this system to track the behaviour of the entire complement of protein complexes from mitotic chromosomes following Seh1 depletion. We initially generated DT40 cells conditionally deficient for Seh1 using the approach previously described (Samejima et al., 2008) in which an intact Seh1 cDNA is under the control of a tetracycline-repressible promoter (Tet-off). Homologous recombination of the gene-targeting construct with the endogenous locus resulted in deletion of the first eight exons of the Seh1 gene (Fig. 5A). Following addition of doxycycline to the medium (denoted as Seh1KO_OFF), Seh1 protein levels expressed from the transgene decreased, becoming undetectable by 36 h (Fig. 5D; Fig. S2B). This was accompanied by a decrease in the growth rate of the Seh1KO_OFF cell cultures (Fig. 5B).

In order to specifically study the mitotic function of Seh1, we modified our conditional Seh1KO cell line and utilised the AID system. In this case, we randomly integrated a plasmid encoding *Oryza sativa* (Os)TIR1 and mAID–GFP–Seh1 into the genome, with the two coding sequences linked by a viral T2A sequence (denoted mAID–GFP–Seh1 KO cells) (Fig. 5C). Expression of the original wild-type Seh1 cDNA was shut off by addition of doxycycline to the medium, and cells were maintained in the presence of doxycycline thereafter, so that the only Seh1 protein expressed was mAID–GFP–Seh1. The expression and correct localisation of mAID-GFP-tagged Seh1 was confirmed by immunofluorescence and western blotting (Fig. 5D; Fig. S2A). Addition of IAA to the medium resulted in rapid depletion of mAID–GFP–Seh1 in 1.5 h (Fig. 5D).

To analyse the mitotic phenotypes associated with Seh1 depletion in DT40 cells, we examined the progression of AIDSeh1 KO cells following release from R03306 arrest in the presence or absence of IAA. R03306 is a reversible CDK1 inhibitor that arrests cells in late G2 (Vassilev et al., 2006). At *t*=0 min following R03306 washout, both control cells (−IAA) and Seh1-depleted cells (+IAA) were in interphase with almost no mitotic cells observed. The mitotic index in control cells increased gradually, peaking at *t*=45 min and then slowly decreasing. At the same time Seh1-depleted cells (+IAA) exhibited a slightly higher mitotic index with an increased number of cells in prometaphase/metaphase, prior to anaphase entry, and in telophase/cytokinesis (Fig. S5A,B). Analysis of metaphase cells in AIDSeh1 KO cells released from R03306 showed an 18% increase in the number of cells with unaligned chromosomes (Fig. S5C,D) and a reduced centromeric localisation of Aurora B (Fig. S5E).

We isolated mitotic chromosomes from Seh1KO and AID Seh1 KO DT40 cells for whole-proteome analysis. Using this approach, we could analyse differences in the composition of the chromosome proteome comparing three different conditions of protein loss: gradual loss of Seh1 over several cell generations due to normal turnover in conventional Seh1KO cells, rapid loss of Seh1 mediated by addition of IAA prior to mitotic entry, or loss of Seh1 from pre-formed mitotic chromosomes mediated by addition of IAA following metaphase arrest with Nocodazole.

Changes in chromosomal protein abundance across all experiments were summarised and presented as a profile plot (Fig. 5E). The grey lines represent levels of all of the 2093 proteins analysed in the experiments. Seh1 (red line) had a similar profile plot in all replicates in the three different experimental set ups, with the most profound depletion seen using the AID system (Fig. 5E). To allow isolation of mitotic chromosomes, a very high mitotic index of >80% of healthy cycling cells is required. Hence, when studying conditional Seh1 KO cells, mitotic chromosome isolation experiments were performed prior to complete Seh1 protein loss (with ∼10–12% protein remaining; Fig. S2C). Cells incubated for longer, to achieve a greater depletion, were not healthy, had a lower mitotic index and could not be used for mitotic chromosome isolation.

**Fig. 6.**
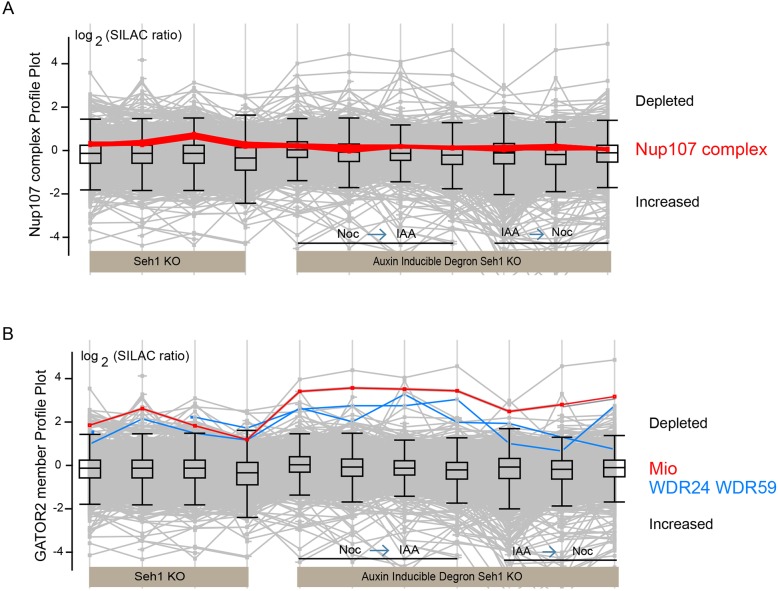
**Seh1 depletion affects the association of the GATOR2 complex with mitotic chromosomes.** (A) Seh1 depletion across different experiments only mildly affects kinetochore levels of Nup107 complex in chromosomes of DT40 cells. Profile plot showing the behaviour of Nup107 complex members (Nup133, Nup107, Nup85, Nup96, Nup160, Elys, Nup37, Nup43, Sec13) (red line). (B) Seh1 depletion strongly affects association of the GATOR2 complex with chromosomes in DT40 cells. Mio is shown as a red line while WDR24 and WDR59 are shown as blue lines.

In our chromosome proteomics experiments, bar plots of mean log_2_H/L ‘stable isotope labelling with amino acids in cell culture’ (SILAC) ratios of individual proteins are used to display the effect of target protein depletion on the levels of each protein in chromosomes. Upwards bars show depleted proteins whereas downwards bars show proteins whose levels on chromosomes are increased. As expected, Seh1 was one of the most highly depleted proteins (Fig. 5F, red bar). Unexpectedly, Mio (also known as MIOS), WDR24 and WDR59 (the other members of the GATOR2 complex) (Bar-Peled et al., 2013; Dokudovskaya et al., 2011; Panchaud et al., 2013a) had similar profiles in virtually all experiments and followed the profile plot of Seh1 (Fig. 5F, grey bar; Fig. 6B). Together, these were the most significantly depleted proteins from chromosomes following Seh1 depletion. Indirect immunofluorescence determination of GATOR2 complex localisation in Seh1–mAIDmC cells, showed a weak association of Mio and WDR24 with mitotic chromosomes in control cells (−IAA) that was reduced following rapid depletion of Seh1 (+IAA) (Fig. S7A,B). At the same time, the total cellular levels of Mio and WDR24 protein were unaffected at early time points (24 h) following doxycycline addition in Seh1KO DT40 cells (Fig. S3D,E) or rapid depletion of Seh1 in AIDSeh1 KO cells (Fig. S3F). Stability of Mio only appeared to be affected after long incubations (13 h) of AIDSeh1 KO cells in the presence of IAA (Fig. S3G). This reveals for the first time that the GATOR2 complex is associated with chromosomes and that this association is specific, as levels of the complex are highly dependent on Seh1. This raises the possibility of some involvement of either Rag1 or mechanistic target of rapamycin (mTOR) with mitotic chromosome structure or function.

Surprisingly, Seh1 depletion only mildly affected chromosomal levels of the Nup107 complex in DT40 cells (Fig. 5F, mid-blue bars). Profile plots of Nup107 complex components (Nup107, Nup133, Nup160, Nup85, Nup96, Nup37, Nup43 and Elys) were nearly identical, consistent with several previous studies, confirming that they also behave as a protein complex in DT40 chromosomes (Harel et al., 2003; Kelley et al., 2015; Lutzmann et al., 2002; Walther et al., 2003) (Fig. 6A). In profile plots, the Nup107 complex showed a consistent, but relatively small depletion from mitotic chromosomes in the absence of Seh1 similar to the small but consistent depletion of Elys detected by indirect immunofluorescence in HCT116 cells (Fig. 4H).

Although we observed a marked loss of chromosomal passenger complex (CPC) localisation at centromeres following Seh1 depletion, we saw only a small reduction in the amount of CPC components associated with mitotic chromosomes (Fig. S2D). This agrees with our previous analysis of chicken kinetochore mutants, in which the association of CPC components with chromosomes was generally unaffected (Samejima et al., 2015a). We believe that this is because quantitative proteomics monitors the behaviour of the entire mitotic chromosome and not only of the centromeric region. It is likely that a dispersed background of CPC distributed across the chromosome arms makes it difficult to see the local decrease that occurs only around centromeres upon Seh1 depletion.

**Fig. 7.**
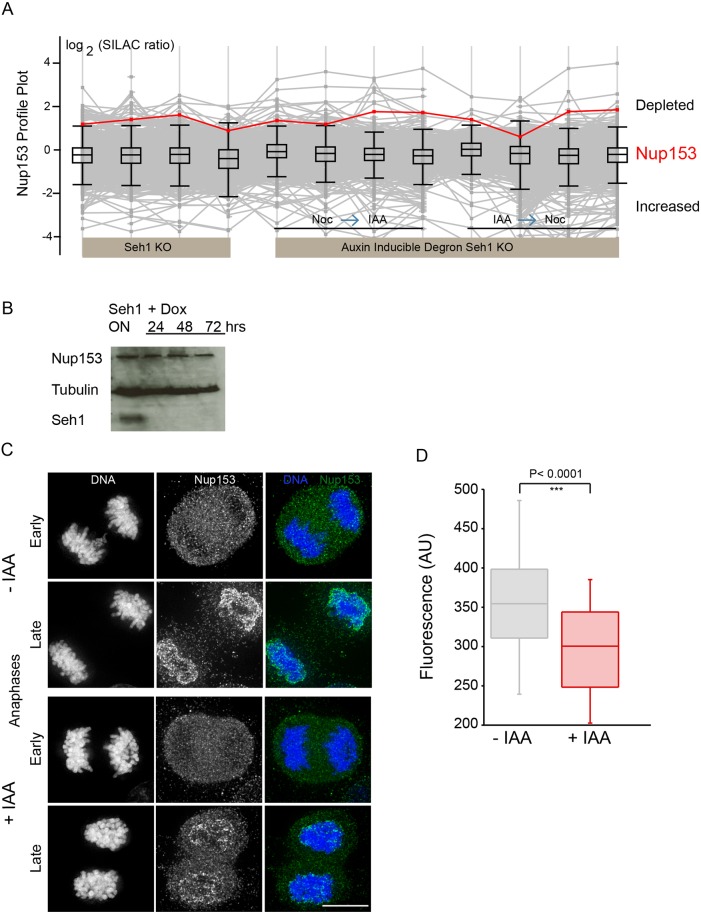
**Seh1 depletion reduces the association of Nup153 with chromosomes.** (A) Profile plot showing the behaviour of Nup153 (red line) following depletion of Seh1 across different experiments. (B) Immunoblots of total cell lysates from Seh1 conditional KO cells in the absence or presence of Dox (24, 48 and 72 h) probed using anti-Nup153 and anti-Seh1 antibodies. Tubulin served as a loading control. (C) HCT116 Seh1–mAIDmC cells at early and late anaphase were fixed and immunostained with anti-Nup153 antibody (green) and for DNA (blue). (D) Quantification of Nup153 levels on anaphase chromosomes in mock (−IAA) (*n*=50) or Seh1-depleted cells (+IAA) (*n*=56) from three independent experiments. Degradation of Seh1 reduces the levels of Nup153 associating with anaphase chromosomes. ***P<0.0001 (two-tailed, unpaired *t*-test). Scale bar: 10 µm.

Analysis of the chromosome proteome revealed a previously uncharacterised metaphase chromosomal pool of Nup153. Nup153 is a nucleoporin of the NPC basket (Ball and Ullman, 2005) that plays an important role in both mitotic progression and NPC assembly in interphase (Mackay et al., 2009; Rines et al., 2008; Vollmer et al., 2015). Nup153 acts in interphase NPC assembly by directing the Nup107 complex to membranes via its Y-complex-binding domain (Vollmer et al., 2015). During mitosis, Nup153 reportedly interacts with Mad1 and affects the spindle checkpoint (Lussi et al., 2010). A reduction of Nup153 levels has been shown to result in an increase in cells with unresolved midbodies following mitosis (Mackay et al., 2009). Surprisingly, the Nup153 profile plot showed the association of Nup153 with chromosomes to be highly dependent on Seh1 protein levels (Fig. 7A). In several experiments, Nup153 was the fifth most depleted protein of 2093 proteins. At the same time, the total cellular levels of Nup153 protein were unaffected following doxycycline addition in Seh1KO DT40 cells (Fig. 7B). Nup153 has been shown by fluorescence microscopy to be recruited to chromatin together with importin B very early during anaphase in human cells (Dultz et al., 2008). However, no major changes in importin B association with mitotic chromatin were detected in DT40 cells depleted of Seh1 (Fig. S1C).

We used human HCT116 Seh1mAIDmC KO cells to ask whether the loss of Nup153 association with chromatin upon Seh1 depletion was conserved across species. Indeed, the levels of Nup153 associated with late anaphase chromosomes were reduced upon addition of IAA (+IAA) to the medium (Fig. 7C,D). This confirmed the DT40 proteomics result and argues that the Seh1-mediated association with mitotic chromosomes is specific. A reduced association of Nup153 with chromatin was also observed following depletion of Seh1 by means of siRNA in HeLa cells (Fig. S3B).

**Fig. 8.**
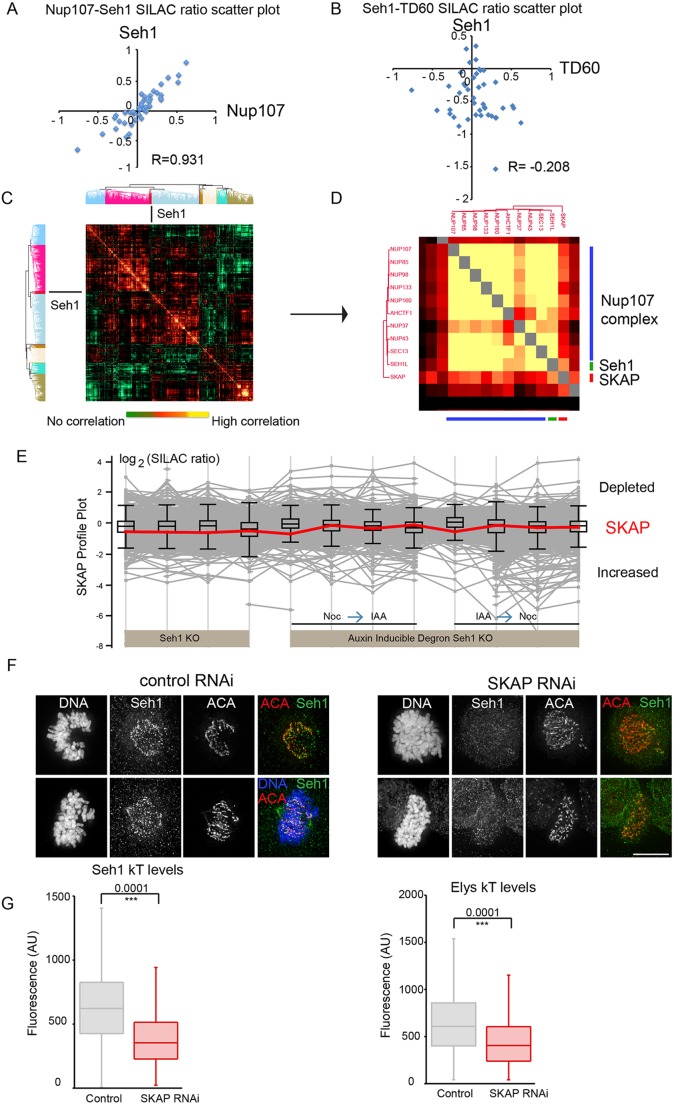
**Seh1 behaviour is highly correlated with the Nup107 complex and the small kinetochore-associated protein SKAP.** (A,B) Scatter plot of H/L ratios (blue diamonds) of Nup107 (A) or TD60 (B) and Seh1 proteins across 38 different chromosome proteomic experiments plotted against each other. The value for Seh1 shows a strong correlation with that for Nup107 protein (Pearson correlation R=0.931) but no correlation with that for TD60 protein (Pearson correlation R=−0.208). (C) Correlation analysis for several chromosomal and kinetochore-associated proteins in this large set of experiments is presented as a color-coded matrix. The position of Seh1 on the matrix is shown by a black line. (D) Magnification of the Seh1 location of the matrix shown in C. (E) Profile plot showing the behaviour of SKAP (red line) following depletion of Seh1 across different experiments. (F) Control and SKAP-depleted cells were fixed and immunostained with anti-Seh1 (green) and -ACA (red) antibodies and for DNA (blue). SKAP depletion affects the kinetochore localisation of Seh1. (G) Quantification of Seh1 and Elys kinetochore levels in control (*n*=34 Seh1, *n*=30 Elys) and SKAP-depleted cells (*n*=35 Seh1, *n*=30 Elys) from three independent experiments. Fluorescence intensities are in arbitrary units (AU). ***P<0.0001 (two-tailed, unpaired *t*-test). Scale bar: 10 µm.

In addition to looking at levels of individual proteins across a range of experiments, an analysis of the correlation of protein behaviour between pairs or groups of proteins across multiple different experiments can confirm the identity of protein complexes and reveal novel functional links between proteins. For example, although Seh1 depletion only mildly affects levels of the Nup107 complex associated with mitotic chromosomes in DT40 cells (Fig. 6A), it has been well documented across a variety of species that Seh1 is part of the Nup107 complex (Kelley et al., 2015). Indeed, over our studies of 12 kinetochore protein knockouts (Samejima et al., 2015a), Seh1 does show a dependency relationship with the Nup107 protein, confirming that it is also part of the complex in mitotic cells of *Gallus gallus* (Fig. 8A).

In a ratio scatter plot, each dot represents SILAC ratios from two proteins from one experiment. Proteins in the same complex show very similar H/L SILAC ratios across experiments. When the SILAC ratios of the two proteins Nup107 and Seh1 from a series of experiments were plotted against each other, an almost straight line was produced. This strong correlation implies a robust interaction between the two proteins. When, as a control, the SILAC ratios of Seh1 and the unrelated protein TD60 were plotted against each other the values were distributed randomly, showing that Seh1 and TD60 are not part of the same complex (Fig. 8B).

To be able to judge the similarity of H/L ratio profiles between pairs of proteins, a Pearson correlation coefficient analysis was performed. In this analysis, co-varying patterns of enrichment or depletion can suggest functional links between proteins. We took advantage of a previous study in which the correlation between every pair of proteins in mitotic chromosomes isolated from cell lines mutant for 12 different kinetochore-associated proteins was calculated, followed by hierarchical clustering analysis was performed and presentation as a heatmap matrix (Samejima et al., 2015a). Each pixel in the matrix represents the correlation coefficient between a pair of proteins (Fig. 8C). Here, we focus on the region of the map containing Seh1 (Fig. 8D).

As expected, Seh1 was identified in a cluster with the Nup107 complex, but surprisingly, it also clustered with the small kinetochore-associated protein SKAP. SKAP is a conserved protein that associates with the spindle and outer kinetochore throughout mitosis (Dunsch et al., 2011; Fang et al., 2009; Schmidt et al., 2010). A profile plot showed that the association of SKAP with bulk chromatin is relatively mildly affected by Seh1 depletion (Figs 8E and 5F). However, SKAP depletion caused a substantial reduction in the amount of Seh1 that was located at kinetochores during mitosis (Fig. 8F). Quantification of levels of Seh1 and Elys at kinetochore following SKAP depletion by means of siRNA in HeLa cells confirmed this novel observation that the Nup107 complex requires SKAP for efficient association with kinetochores (Fig. 8G).

## DISCUSSION

Seh1 is a multifunctional protein that is found in two very different complexes in vertebrate cells. It was first discovered as a nucleoporin – a member of the Nup107 complex, which is a major structural component of the nuclear pore (Belgareh et al., 2001; Bui et al., 2013). Much later, Seh1 was found to be a member of the four-protein GATOR2 complex, which activates mTOR signalling by activating Rag GTPases (Bar-Peled et al., 2013; Panchaud et al., 2013a; Platani et al., 2015). Seh1 also plays an important role in cell division by regulating chromosome alignment and segregation (Platani et al., 2009), acting through the CPC pathway through an unknown mechanism. It is not yet known which of the activities of Seh1 is essential, but mice deficient for Seh1 (International Mouse Phenotype Consortium) are embryonic lethal (Brown and Moore, 2012; de Angelis et al., 2015). Building on previous work, we have here explored the mitotic function of Seh1 by using two different degron AID systems that allow rapid and acute depletion of Seh1 from cells without allowing the cells time to adapt to the different conditions.

Proteomic experiments using Seh1 knockouts yielded several surprising results. We found that Seh1 is not essential for formation of the Y-complex nor for its association with mitotic chromosomes. In Seh1-depleted cells in which residual levels of the protein were less than 5%, the Nup-107 complex still associates with mitotic chromosomes isolated from nocodazole-arrested cells. Y-complex components show a low level of depletion, ranking as the 385th-most depleted of 2093 proteins in this analysis. Thus, we suspect that the mitotic defects discussed below are unlikely to be due to depletion of the Y-complex.

Determination of the proteins most strongly affected by Seh1 depletion yielded two surprising results. First, the GATOR2 complex is associated with mitotic chromosomes, and Seh1 is absolutely required for this association. In Seh1-depleted cells, members of that complex were mostly among the second to sixth most depleted chromosomal proteins, strongly supporting the conclusion that the association of GATOR2 with chromosomes is highly specific and dependent on Seh1. Second, the nucleoporin Nup153, for which there is evidence of a role early in mitosis but which is not known to be associated with kinetochores (Mackay et al., 2009; Rines et al., 2008; Lussi et al., 2010), was surprisingly found to be the seventh most depleted of the ∼2000 chromosomal proteins quantified following Seh1 depletion.

The GATOR2 complex, which is composed of Seh1, Mio, WDR24 and WDR59 (Bar-Peled et al., 2013; Panchaud et al., 2013b), is a positive regulator of the mTOR kinase, which coordinates cell growth and proliferation in response to nutrient supply. Two mTOR complexes have been identified in mammalian cells; mTORC1 and mTORC2 (Guertin and Sabatini, 2007). mTORC1 is a central regulator of cell growth that responds to diverse environmental signals and is dysregulated in several human diseases (Dibble and Manning, 2013; Jewell and Guan, 2013; Laplante and Sabatini, 2012). mTORC1 activation requires Rag GTPases and amino acids to promote mTORC1 translocation to the lysosomal surface (Sancak et al., 2008). Multiple protein complexes regulate mTORC1 including the GATOR1 and GATOR2 complexes. The GATOR1 complex is a GTPase-activating protein (GAP) that inactivates Rag GTPases, thereby inhibiting mTOR activity (Bar-Peled et al., 2013; Panchaud et al., 2013b).

GATOR2 complexes are cytoplasmic and have been localised to lysosomes (Bar-Peled et al., 2013; Wei et al., 2014) in the presence or absence of amino acids (Wolfson et al., 2017). Importantly, we did not detect components of the GATOR1 complex (NPRL3 nor NPRL2), nor Lamp2 or Rag1 (a lysosome marker), Pex5 (a peroxisome marker), nor mTOR kinase in any of our DT40 KO chromosome preparations (although we previously implicated mTOR in the regulation of two mitotic kinases at centrosomes; Platani et al., 2015). The specific Seh1-dependent association of GATOR2 complex members with mitotic chromosomes raises the possibility of this complex having some yet-to-be-discovered function during mitosis. Whether its chromosomal role depends on Rag1 GTPase or on another aspect of mTOR signalling remains to be determined.

NPCs are composed of multiple proteins, termed nucleoporins, that are present in multiple copies (Bui et al., 2013). The nucleoporins Nup153, Nup50 and TPR together comprise the so-called nuclear basket, which attaches to the nucleoplasmic ring of the pore scaffold, formed in part by members of the Nup107 complex. The nuclear basket is thought to be involved in the docking of nuclear export substrates to the nuclear pore prior to transport (Saroufim et al., 2015; Strambio-De-Castillia et al., 2010). Nucleoplasmic Nup153 interacts with and recruits the Nup107 complex to the nuclear envelope, where it functions in interphase NPC assembly (Vollmer et al., 2015). Nup153 has also been found to associate with β-globin-encoding genes and Sox2 in interphase to enable gene regulation (Liu et al., 2017; Toda et al., 2017). Although Nup153 has been implicated in early mitotic progression (Mackay et al., 2009), possibly via interactions with the MAD1 protein (Lussi et al., 2010), no evidence exists for either kinetochore or metaphase chromosome targeting of the protein.

The fact that we observe a significant drop in the levels of chromosome-associated Nup153 upon rapid degradation of Seh1 in three different experimental regimes, suggests the existence of a previously uncharacterised pool of Nup153 that specifically associates with mitotic chromosomes in an Seh1-dependent process. This is not due to a general association of nuclear basket components, as we observed no association of Nup50 with DT40 mitotic chromosomes. We also observed no changes in the interaction of importin B with mitotic chromosomes following Seh1 depletion.

Nup153, importin B and Nup50 were previously reported to be recruited to chromatin very early in anaphase (Dultz et al., 2008) at a time when NPC assembly is initiated on the decondensing chromatin by the Nup107 complex (Gillespie et al., 2007; Rasala et al., 2006). It is interesting to speculate whether chromosome-associated Nup153 might also have a role in Nup107 complex recruitment earlier during mitosis as it does during NPC assembly. Alternatively, the strong dependency of Nup153 on Seh1 for chromosomal localisation raises the possibility that Nup153 might be somehow involved in mitotic GATOR2 localisation or function.

Defining the role of Seh1 and the Nup107 complex in mitosis, and specifically its kinetochore function, has been an important goal in the field since the discovery of the kinetochore localisation of the complex. Previous work based on RNAi depletion studies has demonstrated that the Nup107 complex is required for correct chromosome congression and timely progression through mitosis (Zuccolo et al., 2007). Recently it was shown that Elys, a subunit of the Nup107 complex, mediated the docking of the catalytic subunit of protein phosphatase 1 at kinetochores to direct meiotic chromosome segregation (Hattersley et al., 2016). In addition, RNAi studies have previously suggested that Seh1 regulates mitotic progression by influencing the localisation and activity of the CPC (Platani et al., 2009). Our KO studies confirm this: the mitotic phenotypes observed following Seh1 depletion, which include lengthening the interval from NEBD to anaphase onset, increasing the number of chromosome misalignments, anaphase bridges and multipolar spindles and binucleation, are all regulated by the CPC and Aurora B protein kinase (Vagnarelli and Earnshaw, 2004). As in the Seh1 RNAi studies (Platani et al., 2009), we observed a defect in the centromeric localisation of Aurora B in mid-mitosis following rapid degradation of Seh1.

It has been shown that, to control kinetochore–microtubule attachments, Aurora B phosphorylates and regulates key components of the outer kinetochore–microtubule interface including components of the KMN network (Cheeseman et al., 2006; Ciferri et al., 2008; DeLuca et al., 2006; Wei et al., 2007; Welburn et al., 2010). Indeed, we found decreased phosphorylation of Aurora B substrates Dsn1 and KNL1 to correlate with the reduced centromeric levels of Aurora B. Thus the fine tuning of chromosome segregation events is defective following loss of Seh1. Whether this is a consequence of the role of Seh1 in the Nup107 complex, the GATOR2 complex, or another as-yet-unknown role remains to be determined.

Another unexpected functional link between Seh1 and the kinetochore was revealed when the proteomics studies reported here were combined with results of a previous comprehensive analysis of the kinetochore, and dependencies for association with mitotic chromosomes were determined. This analysis revealed a correlation between the small kinetochore-associated protein SKAP, a conserved component of the vertebrate spindle and outer kinetochore, and the Nup-107 complex. SKAP, together with its partners astrin (also known as SPAG5), dynein light chain LC8 and MYCBP (Kern et al., 2017), localises to spindle microtubules and spindle poles throughout mitosis and to kinetochores from metaphase to telophase. There, it is required for correct spindle assembly and stabilisation of kinetochore–microtubule attachments (Dunsch et al., 2011; Fang et al., 2009; Mack and Compton, 2001; Manning et al., 2010; Schmidt et al., 2010; Thein et al., 2007). Aurora B kinase regulates the localisation of the SKAP–astrin complex to kinetochores, and treatment with an Aurora B inhibitor leads to a small but significant increase in SKAP levels (Manning et al., 2010; Schmidt et al., 2010). A similar increase in SKAP levels at kinetochores was observed here by both quantitative immunostaining and quantitative proteomics of isolated chromosomes following rapid Seh1 degradation. This is consistent with the finding that Aurora B kinase is depleted from centromeres. What was not expected was the reverse dependency in which SKAP depletion led to a marked depletion of the Nup107 complex at mitotic kinetochores.

The complex localisation patterns of SKAP are a result of functional contributions from several interacting partners at kinetochores, including tubulin (Friese et al., 2016). As depletion of KNL1 or the NDC80 complex subunit Nuf2 prevents SKAP localisation to kinetochores (Schmidt et al., 2010; Wang et al., 2012), it appears that SKAP localises peripherally to the NDC80 complex. It was previously shown that targeting of the Nup107 complex at kinetochores depends on CENP-F and the NDC80 complex (Zuccolo et al., 2007). Given the dependencies between SKAP and the Nup107 complex observed here, it will be important to test, in future studies, whether SKAP or astrin might directly or indirectly form a link between the NDC80 complex, Seh1 and the Nup107 complex.

Recent work has highlighted the importance of chemical genetic and proteomic approaches in understanding the formation, function, assembly and dynamics of important nuclear and mitotic complexes, and their role in cell cycle progression. Our data suggest that Seh1 and its interacting partners function in these pathways to mediate proper cell division, nuclear architecture and mTORC1 signalling. Future studies will reveal the role of the Seh1-dependent localisation of GATOR2 and Nup153 to mitotic chromosomes.

## MATERIALS AND METHODS

### Plasmid construction and cell culture

HCT116 cells were grown in McCoy's medium, supplemented with 10% fetal bovine serum, 0.2 mM L-glutamine, 100 U/ml penicillin and 100 µg/ml streptomycin. The NIG272 cell line, an HCT116 cell line stably expressing OsTIR1 was described previously (Natsume et al., 2016). For engineering Seh1–mAIDmC cell line both Seh1 alleles were tagged at the C-terminus of the endogenous locus according to the protocols described in Natsume et al. (2016). C-terminus targeting constructs for the Seh1 gene contained a 5′ homology arm (490 bp), sequence for exon 9 (4 amino acids), mAID tag, mClover, resistance cassette and 3′ homology arm (500 bp). The mAID tag, mClover, hygromycin and G418 resistance cassettes were taken from pMK289 and pMK290 plasmids (Natsume et al., 2016). The guide RNA sequence used was GTAGGCTGCTTCAT.

Seh1 conditional DT40 KO cells (Seh1_KO_OFF and _ON) were created as previously described (Hudson et al., 2003; Samejima et al., 2015b). The endogenous Seh1 gene locus was disrupted and cells depend on the expression of *G. gallus* (Gg)Seh1 cDNA driven by a tetracycline-suppressible promoter. Hence, addition of doxycycline stops the expression of the Seh1 protein. Seh1–mAID–GFP DT40 cells were based on the Seh1-conditional DT40 KO cells. Plasmids encoding Seh1–mAID–GFP and OsTIR1 were randomly integrated into the genome. Expression of the GgSeh1 cDNA fused to the minimal AID tag (mAID) and GFP at its C-terminus was driven by the CMV promoter. The final cell line was cultured in the presence of doxycycline (1 µg/ml) to suppress expression of the non-tagged GgSeh1 protein.

All cell lines used for this study tested negative for mycoplasma contamination.

### RNAi and transfection experiments

cDNA transfections were performed using x-treme Gene (Roche) in HCT116 cells and Neon transfection reagent in DT40 cells. RNAi experiments were performed using annealed siRNA oligonucleotides (Qiagen) using HiPerfect reagent (Qiagen). The SKAP siRNA oligonucleotide sequence was 5′-AGGCUACAAACCACUGAGUAA-3′ (Dunsch et al., 2011). The Seh1 siRNA was as in Platani et al. (2015).

### Preparation of mitotic chromosomes and mass spectrometry

DT40 cells were incubated with 0.5 µg/ml nocodazole for 12–13 h before or after addition of IAA to achieve a minimum mitotic index of >75%. Mitotic chromosomes preparation, mass spectrometry analysis and data processing were carried out as previously described (Samejima et al., 2015a).

### Microscopy and image analysis

For immunofluorescence, 3D data sets were acquired using a CoolSnap HQ cooled CCD camera (Photometrics), on a DeltaVision Spectris microscope (Applied Precision, LLC, WA). Optical sections were acquired every 0.2 µm for fixed cells, or every 0.5 µm for live cells, and 3D data sets were deconvolved using the constrained iterative algorithm (Swedlow et al., 1997; Wallace et al., 2001) implemented in the SoftWoRx software (GE). For live-cell imaging, cells were grown on glass-bottomed Lab-Tek dishes (Nunc), with or without IAA and maintained in a humidified 37°C chamber in CO_2_-independent Phenol Red-free Dulbecco's modified Eagle's medium (DMEM) (Invitrogen). For DNA staining, cells were treated with 100 ng/ml Hoechst 33342 for 30 min, and washed three times with medium prior to imaging. Images were collected using both a 100×/1.4 NA PlanApo objective lens and a 60×/1.4 NA PlanAPO objective. Images were loaded into Photoshop or OMERO (www.openmicroscopy.org) and adjusted for display (Allan et al., 2012).

Quantification of Aurora B, Dsn1, KNL1, Dsn1^S109ph^ and KNL1^Ser24ph^ was carried out as following: deconvolved images were imported into OMERO (Swedlow et al., 2009), and segmentation of centromeric foci [anti-centromere antibodies (ACA); Cy5 reference channel] was performed using the Otsu method, implemented in Matlab, OMEROtools. These segmentation masks were then used to calculate intensities for these bodies in the Aurora B, Dsn1, KNL1, Dsn1^S109ph^ and KNL1^Ser24ph^ channels, and outputted into comma-separated value files for plotting in Excel. The signal within these volumes was quantified, background corrected and represented as the mean fluorescent intensity/pixel. Quantification of the levels of ZW10, SKAP, Elys and Hec1 was similarly calculated, on ∼70 cells per condition.

Quantification of Nup153 and ACA was carried out as following: deconvolved images were imported into OMERO, and segmentation of DNA (Hoechst 33342; 460 nm reference channel) was performed using the Otsu method, implemented in Matlab. The segmentation masks were then used to calculate intensities for these bodies in the Nup153 or ACA channel, and outputted into a comma-separated value file for plotting in Excel. The signal within these volumes was quantified, background corrected and represented as the mean fluorescent intensity/pixel.

### cDNAs, antibodies and immunofluorescence

All fixation, permeabilisation and immunostaining were performed at room temperature. HCT116 cells grown on coverslips were fixed in a 3.7% formaldehyde/PBS solution for 10 min and permeabilised in PBS with 0.5% Triton X-100 for 10 min. DT40 cells grown on poly-lysine coverslips were fixed in a 3.7% formaldehyde/PBS solution for 10 min and permeabilised in PBS with 0.15% Triton X-100 for 5 min. Cells were blocked in 10% normal donkey serum for 1 h at room temperarure prior to antibody incubations. For Nup107, Nup133, SKAP and WDR24 staining, a pre-permeabilisation step with 0.1% Triton X-100 in PHEM buffer for 1 min was included followed by paraformaldehyde fixation. Antibodies used in this study as follows: Elys, 1:1000 (Franz et al., 2007); Lap2b, 1:500 (mouse; cat. no. 611000 BD); mAB414, 1:1000 (mouse; cat. no. MMS-120R, Covance); Seh1, 1:500 (Platani et al., 2009); Tubulin, 1:1000 (mouse; DM1A, cat. no. T6793, Sigma-Aldrich); ACA, 1:300 (human; cat. no. 15-235-0001, Antibodies Incorporated); Aurora B, 1:100 (rabbit; cat. no. ab2254, Abcam); Dsn1, 1:1000; Dsn1^S109ph^, 1:1000; KNL1, 1:1000; KNL1^Ser24ph^, 1:1000 (Welburn et al., 2010); ZW10, 1:100 (rabbit; cat. no. AB21582: Abcam); SKAP, 1:300 (Schmidt et al., 2010); Hec1, 1:300 (mouse; cat. no. ab3613, Abcam); Nup153, 1:200 (mouse; cat. no. ab24700, Abcam); CenpC, 1:100 (mouse; cat. no. ab50974, Abcam); Nup107, 1:100 (rabbit; cat. no. ab178399, Abcam); Nup133, 1:100 (rabbit; cat. no. ab155990, Abcam); Mio, 1:250 (mouse; cat. no. MAB9289-100, R&D Systems), WDR24, 1:250 (rabbit; cat. no. 20778-1-AP, ptglab-Proteintech); GgAuroraB, 1:300 (Xu et al., 2009); and GgCenpT, 1:1000 (Samejima et al., 2018). All affinity purified donkey secondary antibodies (labelled either with Alexa Fluor 488, Alexa Fluor 594 or Alexa Fluor 647) were purchased from Jackson ImmunoResearch. Peroxidase-conjugated donkey anti-rabbit-IgG and goat anti-mouse-IgG antibodies were purchased from Roche. Hoechst 33342 (Life Technologies) was used to stain DNA.

Monastrol (Calbiochem) was used at 100 µM. Nocodazole (Sigma) was used at 0.5 µg/ml for DT40 cells and 100 ng/ml for HCT116 cells. IAA (Fluka-Sigma) was used at 100 µg/ml for DT40 cells and 500 µg/ml for HCT116 cells. R03306 (Sigma) was used at 0.18 µg/ml.

### Statistical analysis

To assess statistical significance, we performed a *t*-test for equal or unequal variances. A *P*<0.05 was considered to be statistically significant. For the box plots, each box encloses 50% of the data with the median value of the variable displayed as a line. The top and bottom of the box mark the limits of 25th and 75th percentile of the variable population. The lines extending from the top and bottom of each box are ×1.5 the box height. Any data points beyond whisker lines are considered outliers.

## Supplementary Material

Supplementary information
