# Supplementary material for: Seh1 targets GATOR2 and Nup153 to mitotic chromosomes
Source: J Cell Sci. 2018 May 1;131(9):jcs213140. doi: 10.1242/jcs.213140 (PMC5992584; doi:10.1242/jcs.213140)
Supplement: Supplementary information [file joces-131-213140-s1.pdf]

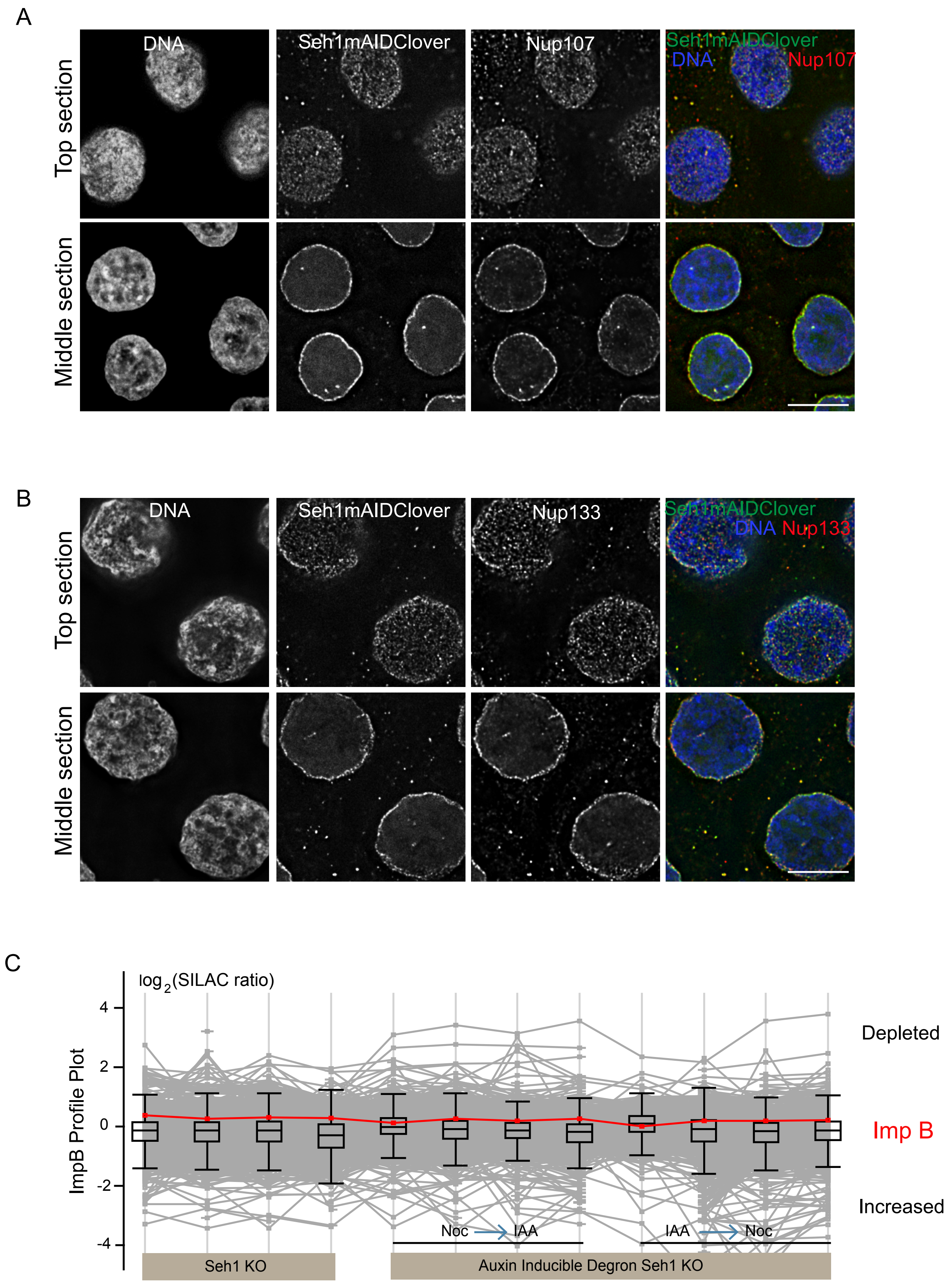

Platani et al. Figure S1

Journal of Cell Science • Supplementary information

Figure 1

(A) and (B) The sub-cellular localisation of endogenous Seh1-mAIDmClover (green) is shown in top or middle nuclear optical sections. Cells were counterstained for Nup107 (red) or Nup133 (also red shown in (B)). DNA is shown in blue. (C) Profile plot showing the behaviour of Importin B (red line) following Seh1 depletion across different experiments. Bar, 10μm.

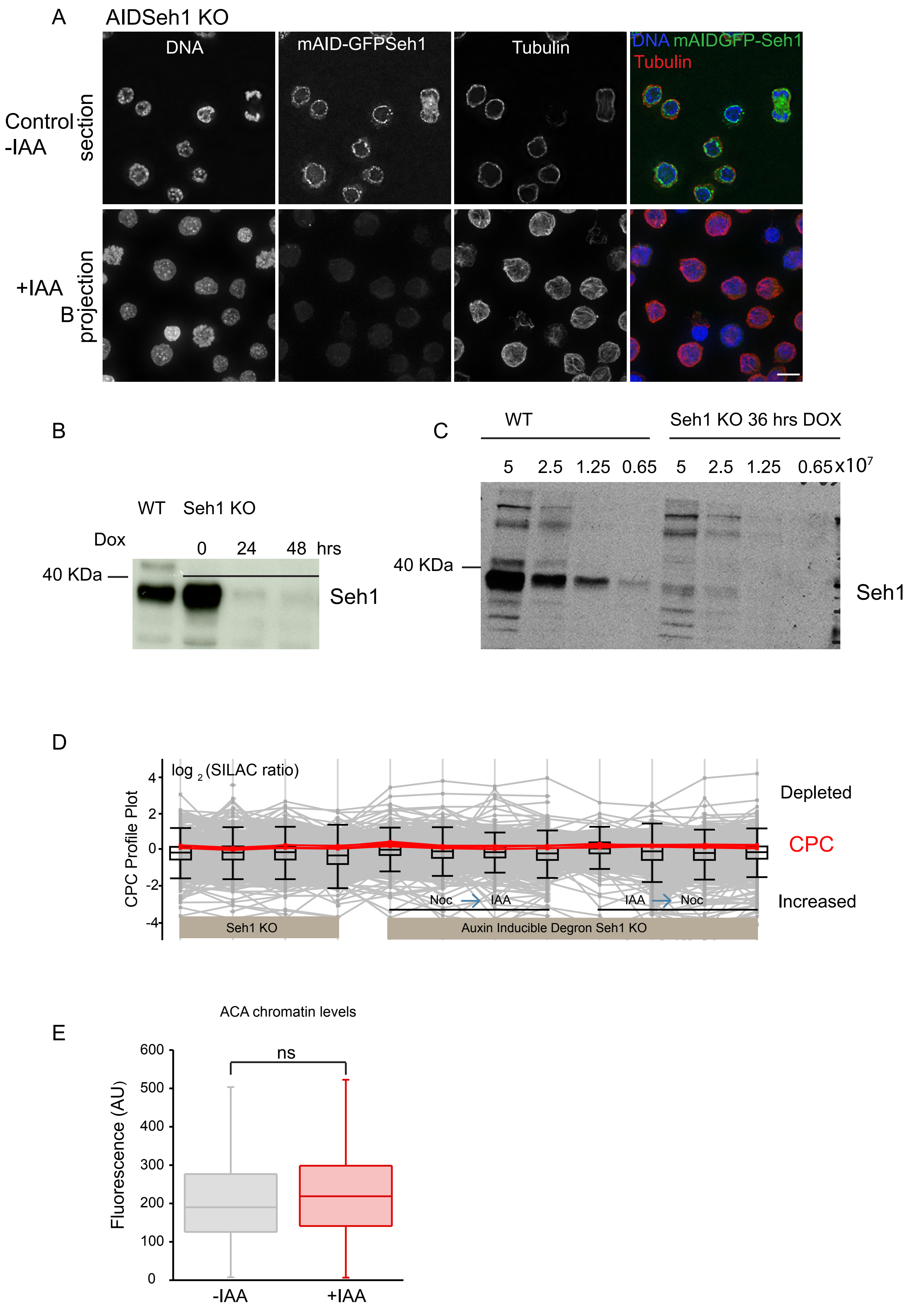

Platani et al. Figure S2

Journal of Cell Science • Supplementary information

Figure 2

(A) The sub-cellular localisation of mAID-CloverSeh1 (green) in DT40 AIDSeh1KO cells is shown in middle nuclear sections in control (–IAA) or Seh1-depleted (+IAA) cells. Cells were counterstained for Tubulin (red). DNA is blue. (B) Immunoblots of total cell lysates of Seh1 conditional KO in the absence or presence of Dox (24 and 48 hrs) probed using anti-Seh1. (C) Immunoblots of total cell lysates of wild type DT40 cells and Seh1 conditional KO cells in the presence of Dox (36 hrs) probed using anti-Seh1. (D) Profile plot showing the behaviour of CPC components (Aurora B, INCENP, Survivin and Borealin) (red lines) following depletion of Seh1 across different experiments). (E) Quantification of ACA levels on chromosomes in mock (-IAA) (n=65), or (+IAA) (n=65) cells from three independent experiments. Statistical significance was determined by a two-tailed, unpaired t-test. Bar, 10μm.

Journal of Cell Science • Supplementary information

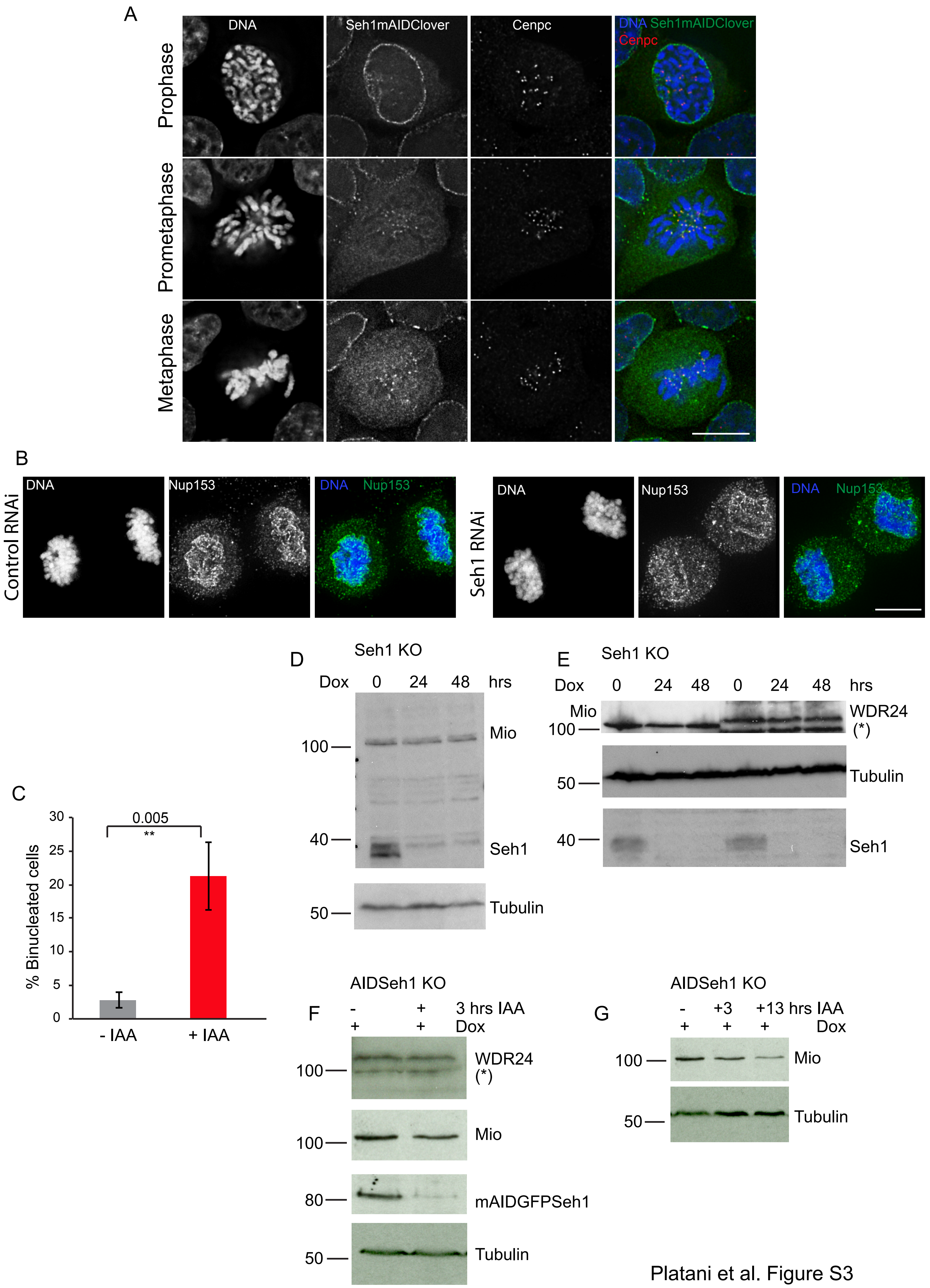

Platani et al. Figure S3

Figure 3

(A) The sub-cellular localisation of endogenous Seh1-mAIDmClover (green) is shown at different mitotic stages. Cells were counterstained for CENP-C (red) to mark centromeres. DNA is shown in (blue). (B) Control and Seh1-depleted cells (RNAi) were fixed and immunostained with anti-Nup153 (green) and DNA (blue). (C) Quantification of binucleated cells in mock treated (-IAA), n=300, Seh1-depleted (+IAA 24hrs), n=300 from four independent experiments. Error bars represent SD. (D,E) Immunoblots of total cell lysates of Seh1 conditional KO in the absence or presence of Dox (24 and 48 hrs) probed using anti-Seh1, anti-Mio, anti-WDR24 and anti-Tubulin. (F,G) Immunoblots of total cell lysates of Seh1-mAIDmC cell line in the absence (-IAA) or presence (+IAA) probed using anti-Seh1, anti-Mio, anti-WDR24 and anti-Tubulin. Tubulin serves as a loading control. (*) nonspecific band. Statistical significance was determined by a two-tailed, unpaired t-test.Bar, 10μm.

Journal of Cell Science • Supplementary information

A

Seh1mAIDC
Control (- IAA)

B

Seh1mAIDC
+ IAA

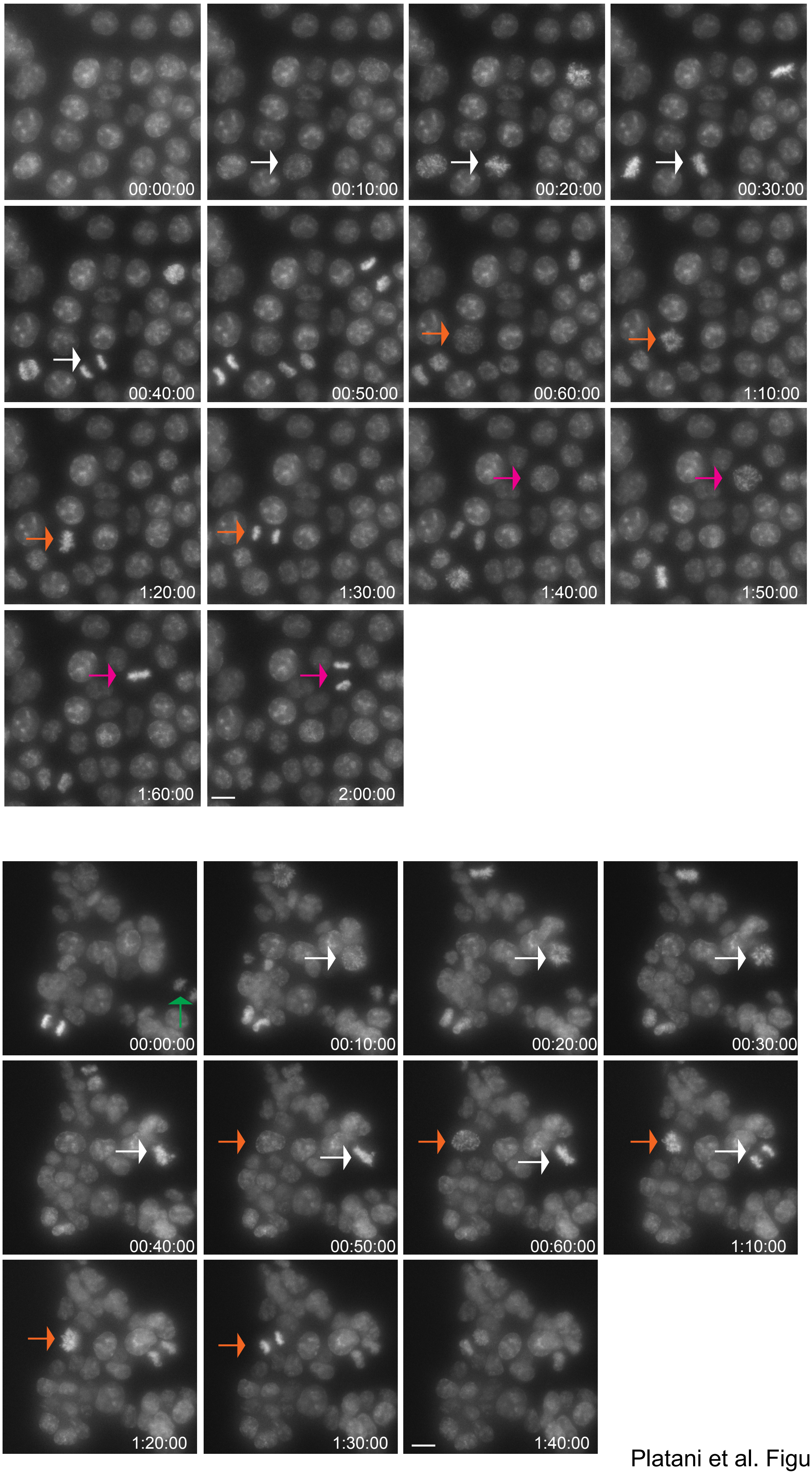

Platani et al. Figure S4

Journal of Cell Science • Supplementary information

Figure 4

Selected maximum intensity projections from time-lapse images of control (-IAA) Seh1-mAIDmC cells (A), (arrows point to mitotic cells) or Seh1-mAIDmC cells following addition of IAA (+IAA) (B). Arrows point to mitotic cells or chromatin bridges. DNA stained with Hoechst33342. Images were collected every 10 min over 3 hrs. Numbers indicate time in hrs:minutes:seconds. Bar, 10μm.

Journal of Cell Science • Supplementary information

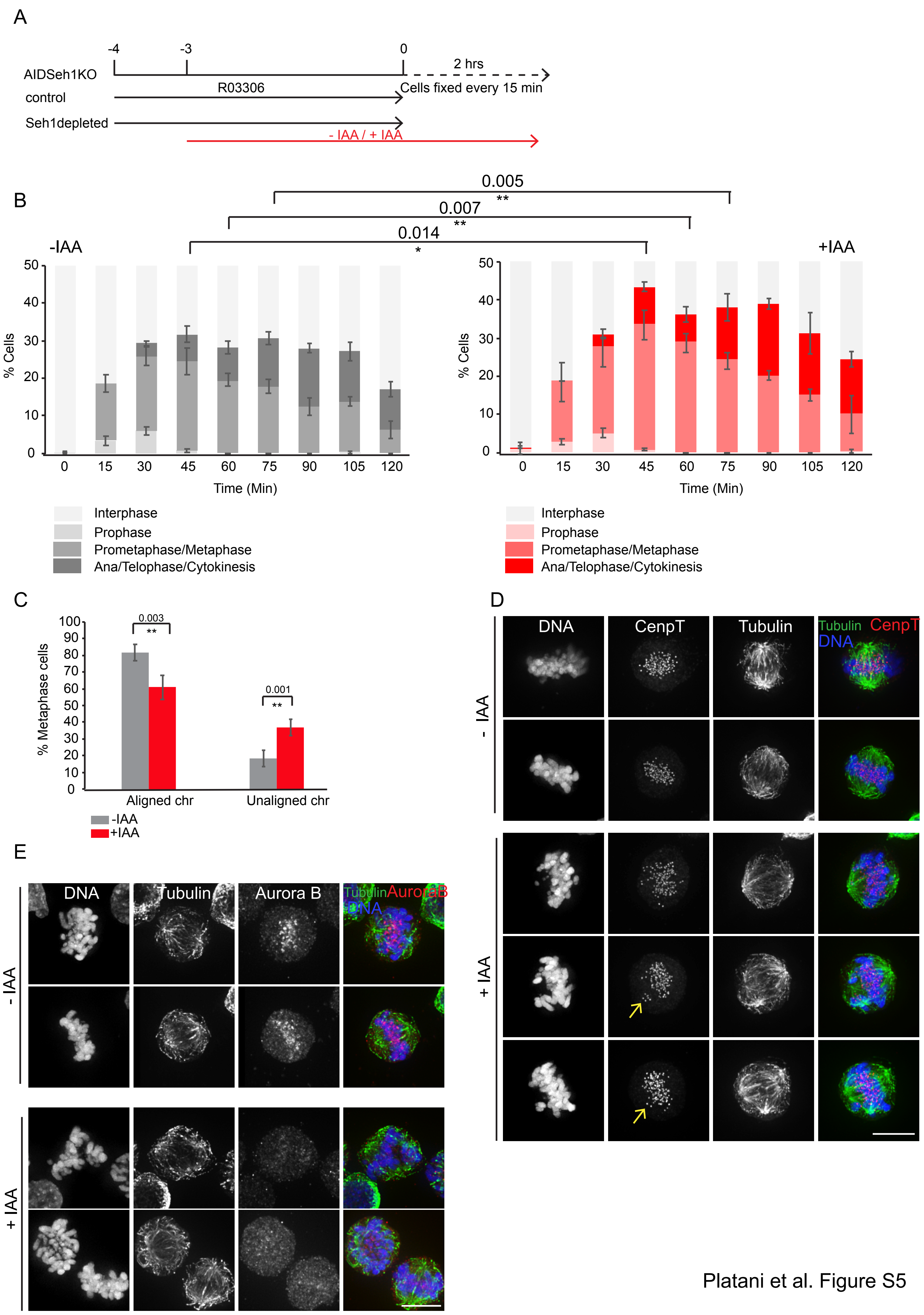

Platani et al. Figure S5

Journal of Cell Science • Supplementary information

Figure 5

(A) Schematic diagram of AIDSeh1 KO DT40 cell line synchronisation with R03306 prior to -/+ IAA addition. AIDSeh1 KO cells were mock incubated (-IAA) or auxin treated (+IAA) for 3 hrs. The drug was washed out with fresh medium prior to release in -/+IAA. Cells were fixed at the indicated time points and immunostained with anti-tubulin (green), anti-CENP-T (red) and DNA (blue). (B) Quantitation of mitotic stages in mock (-IAA) (grey bars) and Seh1-depleted (+IAA) cells (red bars) at the indicated time points after R03306 release. n=400 (-IAA) and n=400 (+IAA) cells from four independent experiments. (C) Quantitation of aligned and unaligned chromosomes in mock (-IAA) (grey bars) and Seh1-depleted (+IAA) cells (red bars) from AIDSeh1 KO cells following R03306 release at T=45 min. n=142(-IAA) and n=193(+IAA) cells from four independent experiments. (D, E) Representative images of control (-IAA) and Seh1-depleted (+IAA) treated AIDSeh1 KO cells following R03306 release at T=45 min were fixed and immunostained with anti-Aurora B (red), anti-CENP-T (red), α-Tubulin (green), and DNA (blue). Statistical significance was determined by a two-tailed, unpaired t-test. Error bars represent SD. Bar, 5 μm.

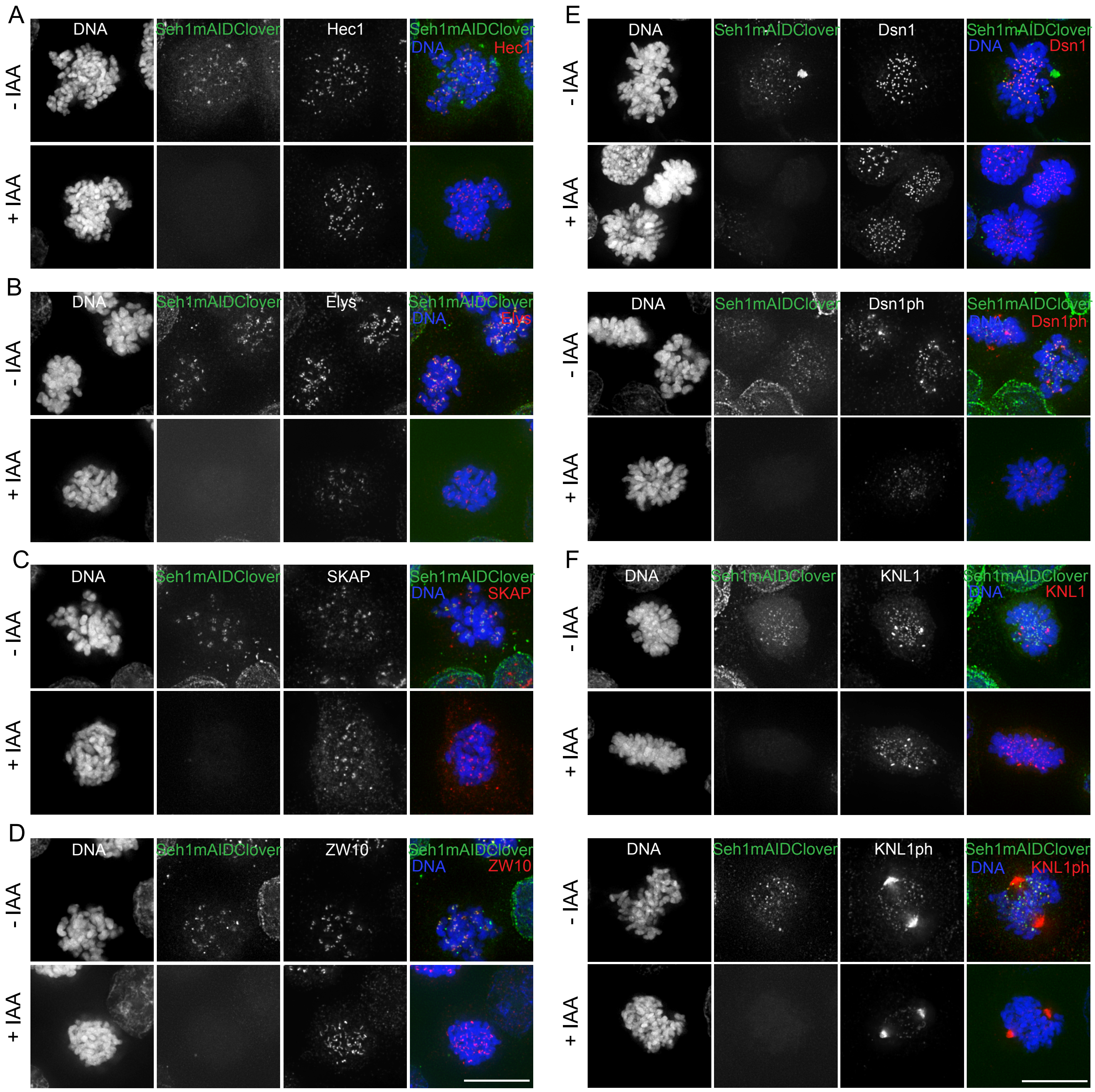

Platani et al. Figure S6

Figure 6

Mock (-IAA) and Seh1-depleted (+IAA) treated Seh1-mAIDmC cells were fixed and immunostained with anti-Hec1 (red) (A), anti-Elys (red) (B), anti-SKAP (red) (C), anti-ZW10 (red) (D), anti-Dsn1 (red) and anti-Dsn1ph (red) (E), anti-KNL1 (red) and anti-KNL1ph (red) (F) DNA, blue. Seh1-mAIDmClover is green. Bar, 10μm.

Journal of Cell Science • Supplementary information

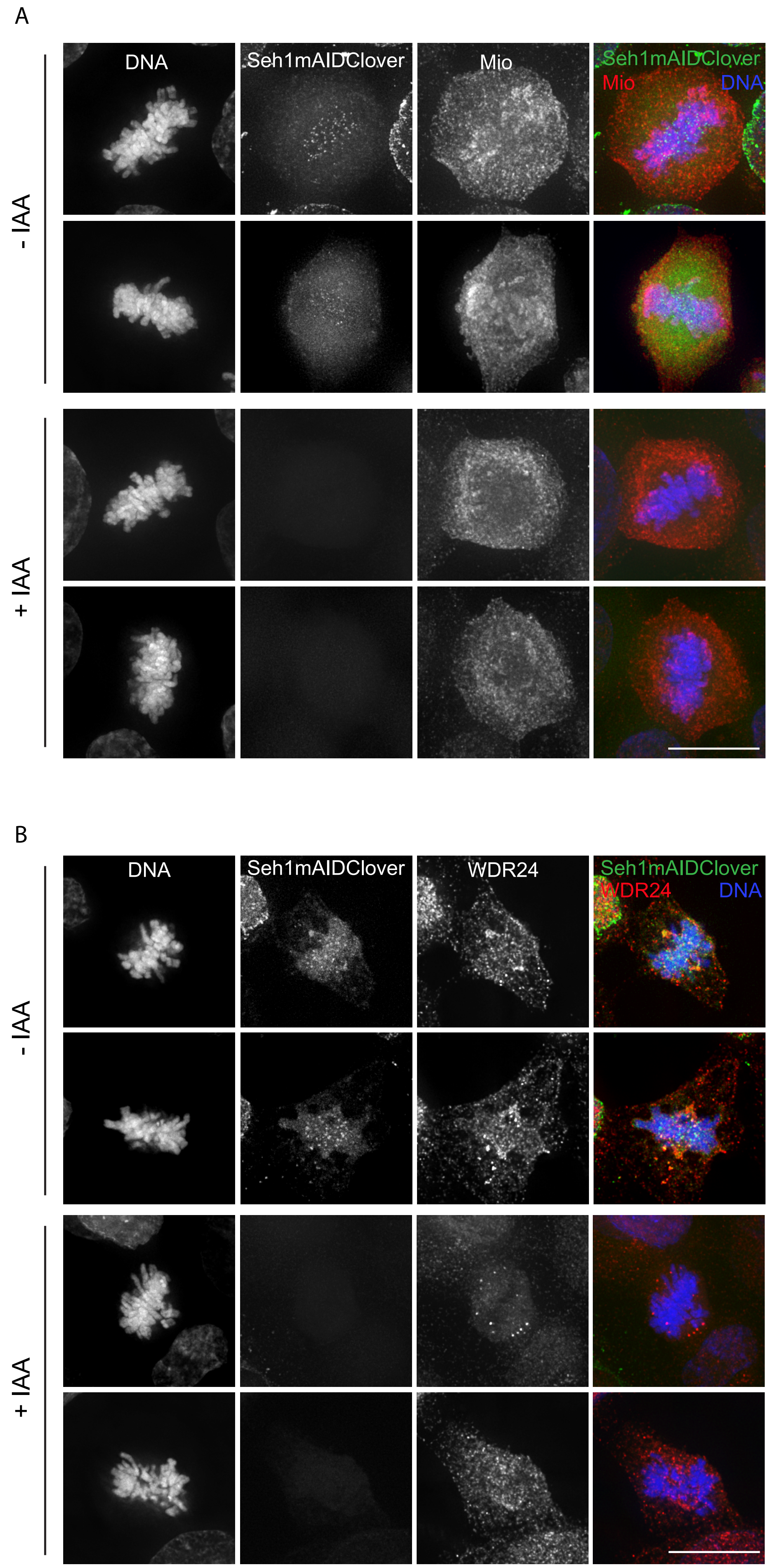

Platani et al. Figure S7

Journal of Cell Science • Supplementary information

Figure 7

Mock (-IAA) and Seh1-depleted (+IAA) treated Seh1-mAIDmC cells were fixed and immunostained with anti-Mio (red) (A) and anti-WDR24 (red) (B), DNA, blue. Seh1-mAIDmClover is green. Bar, 10μm.

Journal of Cell Science • Supplementary information
